# Mitochondrial DNA copy number as a genetic determinant of renal function: insights from bidirectional Mendelian randomization

**DOI:** 10.1080/0886022X.2025.2542522

**Published:** 2025-08-10

**Authors:** Suwei Wang, Yuanjing Yang, Zeyu Pang, Yidan Li, Ke Li, Yan Sun, Jurong Yang

**Affiliations:** ^a^ Department of Nephrology, The Third Affiliated Hospital of Chongqing Medical University, Chongqing, China; ^b^ Department of Orthopedics, The Third Affiliated Hospital of Chongqing Medical University, Chongqing, China; ^c^ Core Research Laboratory, the Second Affiliated Hospital, School of Medicine, Xi’an Jiaotong University, Xi’an, Shanxi, People’s Republic of China

**Keywords:** Mitochondrial DNA copy number, renal function, mendelian randomization, genetic causality

## Abstract

**Background:** Observational studies suggest a correlation between mitochondrial DNA copy number (mtDNA-CN) and renal function; however, the causality remains uncertain. This study employed a two-sample bidirectional Mendelian randomization (MR) analysis to investigate the genetic causal relationship between mtDNA-CN and renal function. **Methods:** Genome-wide association study (GWAS) data for mtDNA-CN were obtained from the UK Biobank (*n* = 395,718), with renal function data primarily sourced from the CKDGen consortium and FinnGen studies. Four MR methods were employed, using inverse variance weighting as the primary approach, complemented by weighted median, MR Egger, and MR-PRESSO for sensitivity analyses. Multivariable MR (MVMR) assessed result robustness. Reverse MR treated renal function as the exposure. Validation was performed using additional mtDNA-CN GWAS data from the CHARGE UK Biobank (*n* = 465,809). **Results:** Forward MR analysis demonstrated a positive association between genetically predicted mtDNA-CN and estimated glomerular filtration rate (eGFR) [odds ratio (OR) = 1.007, 95% CI 1.003–1.012, *p* = 0.003]. MVMR suggested weaker evidence after adjusting for neutrophil count. Reverse MR revealed causal associations of urinary albumin-creatinine ratio (OR = 0.958, 0.923–0.994, *p* = 0.023) and microalbuminuria (OR = 0.981, 0.965–0.997, *p* = 0.021) with mtDNA-CN, though these effects were non-significant after multiple testing correction. Sensitivity and validation analyses confirmed robust. The findings from validation analyses were consistent. **Conclusion:** Our study suggests a potential causal association between mtDNA-CN and eGFR. However, the impact of confounding factors and the absence of consistent associations with other renal function markers underscore the necessity for further research to clarify the role of mtDNA-CN in renal function.

## Introduction

Mitochondrial DNA copy number (mtDNA-CN) is a measure of mitochondrial abundance, with each mitochondrion containing multiple copies of DNA. Proteins encoded by mtDNA are essential for maintaining mitochondrial function [1,2]. Changes in mtDNA-CN reflect the functional status of mitochondria [3]. MtDNA-CN can influence cellular energy metabolism and stress responses by regulating mitochondrial biogenesis, clearance, and damage repair mechanisms [4]. In 1990, Douglas Wallace proposed that mtDNA damage leads to mitochondrial dysfunction and metabolic defects, forming the basis for many common aging and degenerative diseases [5,6]. Increasing evidence suggests that a decreased mtDNA-CN is associated not only with the aging process [7], but also with the progression of various chronic diseases, such as cancer [8], mental disorders [9,10], and cardiovascular diseases [11]. The kidney is a vital organ that requires a substantial amount of energy to actively regulate the body’s metabolism, plasma hemodynamics, electrolyte and water homeostasis, nutrient reabsorption, and hormone secretion [12]. Mitochondria are increasingly recognized as key players in genetic and acquired renal diseases [13]. In 1995, Rotig et al. first reported an association between mtDNA deletions and renal involvement in primary hereditary mitochondrial diseases [14]. In recent years, the correlation between changes in mtDNA-CN in blood and renal function impairment has garnered widespread attention [15,16]. In 2009, Malik et al. found that patients with diabetic nephropathy had significantly increased average mtDNA values in their peripheral blood, 2–4 times higher than those in diabetic patients without kidney disease (*p* < 0.05) [17]. This is the first observational study to report an association between mtDNA-CN and kidney disease. Multiple studies have also found associations between mtDNA-CN and various renal injury markers [18–20]. However, owing to the inherent biases from inverse causation and confounding factors in observational studies, causal relationships cannot be easily inferred. To date, it remains unclear whether a causal genetic relationship exists between the mtDNA-CN and renal function.

Mendelian randomization (MR) analysis is derived from Mendel’s laws of inheritance, and focuses primarily on how genetic variations influence the occurrence and development of diseases [21,22]. This method uses genetically determined variations randomly allocated at the chromosomal level as instrumental variables to estimate the causal effects of exposure on the outcomes [23,24]. The greatest advantage is its ability to avoid potential bias from confounding factors and inverse causation [25]. Combining mtDNA-CN with mendelian analysis allows for a more in-depth exploration of the specific impacts of genetic variations on kidney health. This combined research not only helps elucidate the complex genetic mechanisms of kidney diseases but may also provide new ideas and strategies for personalized medicine. This study employed a two-sample MR analysis using data from genome-wide association studies (GWAS) to investigate the causal relationship between mtDNA-CN and renal function.

## Materials and methods

### MR design

**Figure 1.**
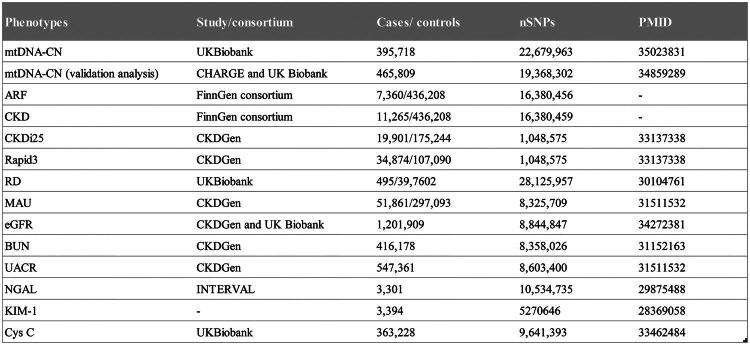
Data sources of the used genome-wide association study in the study. MtDNA-CN: mitochondrial DNA copy number; IV: instrumental variable; ARF: acute kidney injury; CKD: chronic kidney disease; CKDi25: progression of CKD; Rapid3: rapid decline in eGFR; RD: renal dialysis; MAU: microalbuminuria; eGFR: estimated glomerular filtration rate; BUN: blood urea nitrogen; UACR: urine albumin-to-creatinine ratio; NGAL: neutrophil gelatinase-associated lipocalin; KIM-1: kidney injury molecule-1; CysC: cystatin C.

Mendelian analysis is based on three assumptions: (1) relevance assumption: single nucleotide polymorphisms (SNPs) are associated with the exposure; (2) independence assumption: SNPs are not associated with other variables related to the exposure-outcome; (3) exclusion restriction assumption: SNPs influence the outcome only through the exposure and not through other pathways [21,25]. This study explores the causal relationship between mtDNA-CN and renal function based on summarized data from GWAS. Renal function is typically measured by the clearance rates of small molecules such as creatinine and urea; however, with the deepening of research in the field of nephrology, indicators reflecting kidney damage have also become increasingly diverse. According to the Kidney Disease Improving Global Outcomes (KDIGO) guidelines [26], we included 12 phenotypes of renal function: acute kidney injury (ARF), chronic kidney disease (CKD), estimated glomerular filtration rate (eGFR), blood urea nitrogen (BUN), progression of CKD (CKDi25), rapid decline in eGFR (Rapid3), renal dialysis (RD), microalbuminuria (MAU), urine albumin-to-creatinine ratio (UACR), neutrophil gelatinase-associated lipocalin (NGAL), kidney injury molecule-1 (KIM-1) and cystatin C (CysC). All the data used in this study are publicly available, and detailed information is provided in Figure 1. This study is reported in accordance with the Strengthening the Reporting of Observational Studies in Epidemiology Using Mendelian Randomization (STROBE-MR) reporting guideline [27]. All included studies received approval from their respective academic ethics review boards and written informed consent was obtained from each participant [28].

### GWAS summary statistics of mtDNA-CN

GWAS statistics on mtDNA-CN were collected by sourcing instrumental variables (IV) [29] from 395,781 UK Biobank (UKB) participants. The estimating of mtDNA-CN was based on a new method of AutoMitoC pipeline developed by Chong et al. [30], which provides a comprehensive genetic assessment of mtDNA-CN and rapid and accurate estimation of mitochondrial DNA concentration in blood samples. In the validation study, GWAS statistics for mtDNA-CN was extracted from Longchamps et al. [31] involving 465,809 Caucasian individuals from the Cardiovascular and Aging Study cohorts of the Genomics Epidemiology Alliance and UKB.

### GWAS summary statistics of renal function

We conducted a comprehensive search of currently published GWAS summary statistics related to kidney diseases, including 12 renal function outcomes. GWAS estimated statistics for ARF (7360 cases and 436,208 controls) and CKD (defined as renal function impairment lasting three months or longer due to chronic renal injury, including 11,265 cases and 436,208 controls) were derived from the FinnGen database (https://r11.finngen.fi/). Summary statistics for eGFR [32] from GWAS were obtained from a meta-analysis by the CKDGen Consortium [33] and, which used blood creatinine measurements and estimated GFR using the CKD-EPI formula (*n* = 1,201,909). BUN [34] statistics were obtained from a large-scale GWAS meta-analysis by Wuttke et al. which was limited to individuals of European descent, including 416,178 individuals. UACR [35] (*n* = 547,361, median in each study 7.5 mg/g) and MAU [35] (51,861 cases and 297,093 controls) statistics were extracted from a cross-ethnic GWAS meta-analysis led by Teumer, mainly involving individuals of European descent. To study the dynamic effects of mtDNA-CN on kidney damage, we included a genome-wide association meta-analysis of rapid eGFR decline conducted by Gorski, featuring two endpoints: Rapid3 (defined as a decline in eGFR of more than 3 mL/min/1.73 m2 per year, including 34,874 cases and 107,090 controls) and CKDi25 (defined as a decline in eGFR to 25% below baseline, with a final eGFRcrea below 60 mL/min/1.73 m2, including 19,901 cases and 175,244 controls) [36]. The datasets for eGFR, BUN, UACR, MAU, Rapid3, and CKDi25 were obtained from the CKDGen database (http://ckdgen.imbi.uni-freiburg.de/). GWAS summary statistics for RD [37] were primarily derived from the UK Biobank (UKB), involving 495 cases and 397,602 controls. GWAS data for KIM-1 [38] and NGAL [39] were obtained from 3394 and 3301 European samples, respectively. Cystatin C [40] GWAS data were sourced from the UKB, covering 342,399 individuals of European, 6,015 individuals of African, and 7338 individuals of South Asian descent (total *n* = 363,228).

### Extraction of instrumental variables

**Figure 2.**
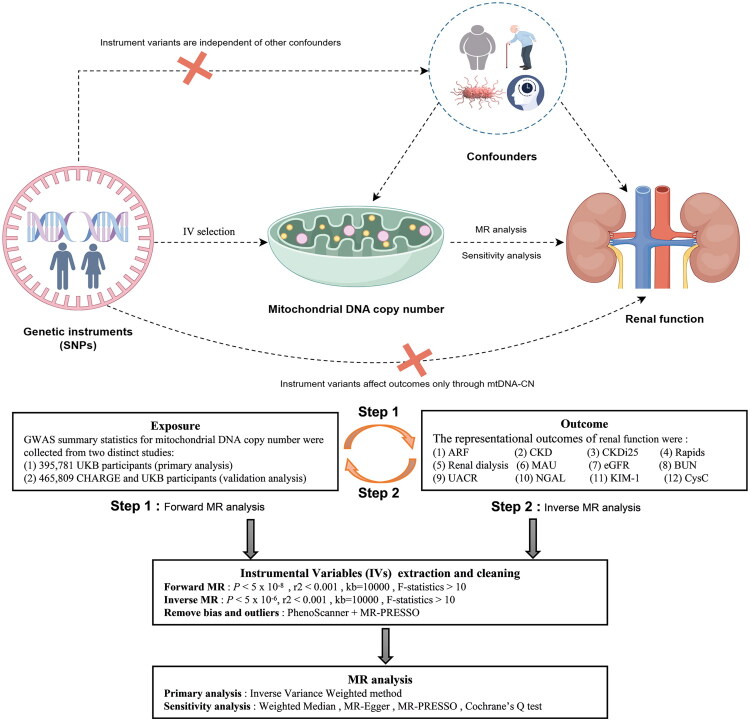
Overview of study design of the bidirectional Mendelian randomization framework used to investigate the causal effect of mitochondrial DNA copy number on renal function. GWAS: genome-wide association study. IV: instrumental variable.

Genetic association IV must adhere strictly to the principles of MR. The research framework of this study is shown in Figure 2. In the forward MR analysis, the genome-wide significance threshold for SNPs associated with mtDNA-CN is set at *p* < 5 × 10^−8^. For the reverse MR analysis, due to limitations on the number of coordinating SNPs, the threshold for renal function is adjusted to *p* < 5 × 10^−6^. Furthermore, the method for selecting SNPs should ensure their independence from each other, thereby minimizing the potential confounding effects caused by linkage disequilibrium (LD). SNPs were assessed for independence with R^2^ < 0.001 (aggregation window size = 10,000 kb), retaining only the SNPs with the lowest p-values, and subsequently excluding those with a minor allele frequency (MAF) ≤ 0.01. In cases where palindromic SNPs exist, forward-strand alleles are inferred using allele frequency information. Only SNPs with an F-statistic > 10 were retained, eliminating weak instrumental variables that may have introduced bias into the results [41]. To further exclude biased SNPs, we searched the PhenoScanner [42] database for genotype-phenotype associations of the SNPs, eliminating potential confounding factors, such as obesity, aging, and diabetes, to ensure that SNPs with a direct impact on the results were not included in the study. Fortunately, none of the SNPs showed any indications of exclusion. Additionally, SNPs with pleiotropic effects can introduce bias in inverse variance weighted (IVW) analysis and lead to a loss of statistical power. Therefore, we utilized MR-PRESSO [43] to identify pleiotropic outliers and removed them before conducting the MR analysis to obtain the optimal SNPs.

### Statistical analysis

This study employed four methods to analyze the causal relationship between exposure and outcome: IVW, MR-Egger [44], weighted median estimation (WM) [24,45], and MR-PRESSO [43]. The results of the IVW serve as the primary reference for our investigation of causal associations, with the advantage of variance minimization. If the causal relationships identified using the other three analytical methods were consistent with the IVW results (*p* < 0.05), this would significantly enhance our confidence in establishing causal relationships. Cochrane’s Q was used to assess the heterogeneity of SNP estimates, with *p* < 0.05, considered significant in the heterogeneity test. To test for horizontal pleiotropy and outliers, MR-Egger and MR-PRESSO were used to conduct sensitivity analyses from different perspectives. In the MR-Egger analysis, the p-value of the intercept was used to evaluate the horizontal pleiotropy (*p* < 0.05). MR-PRESSO analysis was performed to identify and correct for potential pleiotropic outliers. The leave-one-out method was implemented to determine whether any single SNP drove the estimates by sequentially excluding each SNP. MR analysis is presented as odds ratios (OR) accompanied by 95% confidence intervals (CI). To reduce bias from false-positive evidence in the results, we applied the Benjamini and Hochberg (BH) method for false discovery rate (FDR) correction to all *p*-values. A *p*-value ≤ 0.05, which did not reach the FDR correction significance threshold (*p* ≤ 0.05), indicated potential causal association evidence. To evaluate the independent association between mtDNA-CN and renal function while accounting for potential confounding effects, we conducted Multivariable Mendelian Randomization (MVMR) analysis, adjusting for blood cell counts (neutrophil count, lymphocyte count, monocyte count, platelet count) and blood lipids (total cholesterol, total triglycerides, apolipoprotein A1, apolipoprotein B, apolipoprotein E) (GWAS IDs provided in the Supplementary Table S9). In the MVMR analysis, the same genetic variants used for each exposure in the univariable analysis were integrated into a multivariable genetic instrument, and a multivariable regression model was fitted to assess the independent causal effect of mtDNA-CN on renal function under the influence of different confounding factors [46,47]. All MR results were analyzed using the Two Sample MR R package (version R4.4.1).

## Results

### Association of mtDNA copy number with renal function

A total of 6392 SNPs were obtained with mtDNA-CN at genome-wide significance. After removing linkage disequilibrium and weak instrumental variables, 64, 64, 10, 10, 64, 50, 57, 62, 62, 18, 65 and 43 SNPs associated with mtDNA-CN were selected from the GWAS database of ARF, CKD, CKDi25, Rapid3, RD, eGFR, BUN, UACR, MAU, CysC, NGAL and KIM-1 (Supplementary Table S1). The IVW analysis showed that the mtDNA-CN was positively associated with eGFR [odds ratio (OR)=1.007, 95%CI: 1003–1.012, *p* = 0.003, FDR. *p* = 0.036]. No significant genetic causal associations between mtDNA-CN with other phenotypes of renal function.

**Figure 3.**
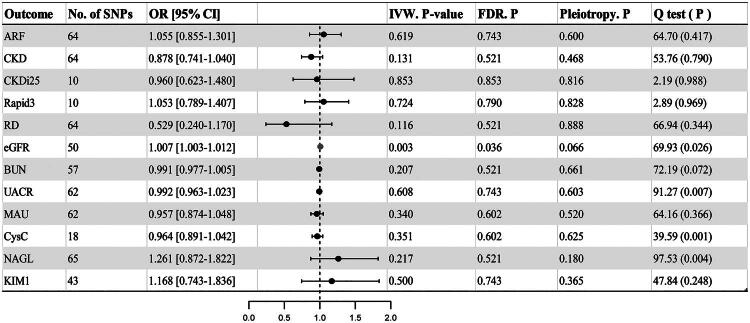
Causal effects of mtDNA-CN on renal function. No. of SNPs: number of single nucleotide polymorphism; or: Odds ratios, represent the odds ratios per 1-standardized unit (in SD unit) increase in the mtDNA copy number; 95% CI: 95% confidence interval; IVW: inverse variance-weighted; FDR: false discovery rate; pleiotropy: the MR-Egger regression method was used to identify whether there are pleiotropic effects; Q test: the Cochran’s Q statistics were used to reflect heterogeneity between the SNP-specific estimates.

**Figure 4.**
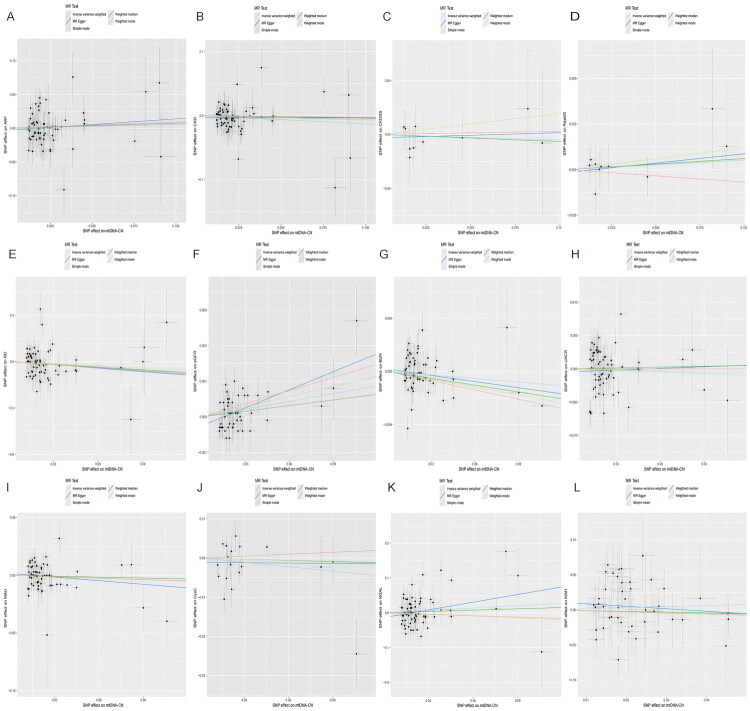
The forward MR analyses: Scatter analyses. (A) ARF, (B) CKD, (C) CKDi25 (D) Rapid3, (E) RD, (F) MAU, (G) eGFR (H) BUN, (I) UACR, (J) Cys C, (K) NGAL, (L) KIM-1. The four methods applied in the current manuscript were all depicted. Lines in black, red, green, and blue represent IVW, MR‐Egger, weighted median, and weight mode methods.

In the sensitivity analyses, there was no indication of horizontal pleiotropy in the MR-Egger intercept for all outcomes (Figure 3 and Supplementary Table S2). The heterogeneity of mtDNA-CN for eGFR, UACR, NGAL, and CysC was statistically significant according to Cochrane’s Q test. Figure 4, Supplementary Figures S1 and S2 summarize the results of sensitivity analysis (scatter plots, funnel plots, and leave-one-out analysis). Results from verification analyses supported a strong causal association between mtDNA-CN and eGFR (Supplementary Tables S3 and S4, Figure S3, S4 and S5).

**Figure 5.**
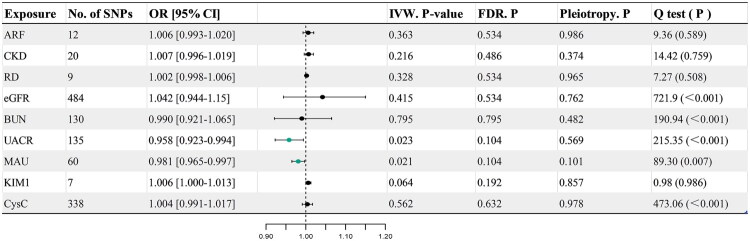
Causal effects of renal function on mtDNA-CN. No. of SNPs: number of single nucleotide polymorphism; or: Odds ratios, represent the odds ratios per 1-standardized unit (in SD unit) increase in the mtDNA copy number; 95% CI: 95% confidence interval; IVW: inverse variance-weighted; FDR: false discovery rate; pleiotropy: the MR-Egger regression method was used to identify whether there are pleiotropic effects; Q test: the Cochran’s Q statistics were used to reflect heterogeneity between the SNP-specific estimates.

### Association of renal function with mtDNA copy number

In reverse MR analysis, due to the limited number of coordinating SNPs for CKDi25, Rapid3, and NGAL, there were still not enough SNPs for follow-up studies, even after we relaxed the screening criteria; therefore, we abandoned the reverse MR analysis for these three outcomes. In reverse MR analyses, 12, 20, 9, 484, 130, 135, 60, 7 and 338 SNPs linked to ARF, CKD, RD, eGFR, BUN, UACR, MAU, KIM-1 and CysC were screened from the mtDNA CN GWAS database (Supplementary Table S5).

The results of the reverse MR analysis are shown in (Figure 5 and Supplementary Table S6). There is no strong evidence that ARF, CKD, eGFR, and BUN are related to mtDNA-CN, whereas UACR (OR = 0.958, 95%CI: 0.923–0.994, *p* = 0.023, FDR.*p* = 0.104) and MAU (OR = 0.981, 95%CI: 0.965–0.997, *p* = 0.021, FDR.*p* = 0.104); however, the P-values of both values did not reach the adjusted significance threshold (*p* ≤ 0.05).

**Figure 6.**
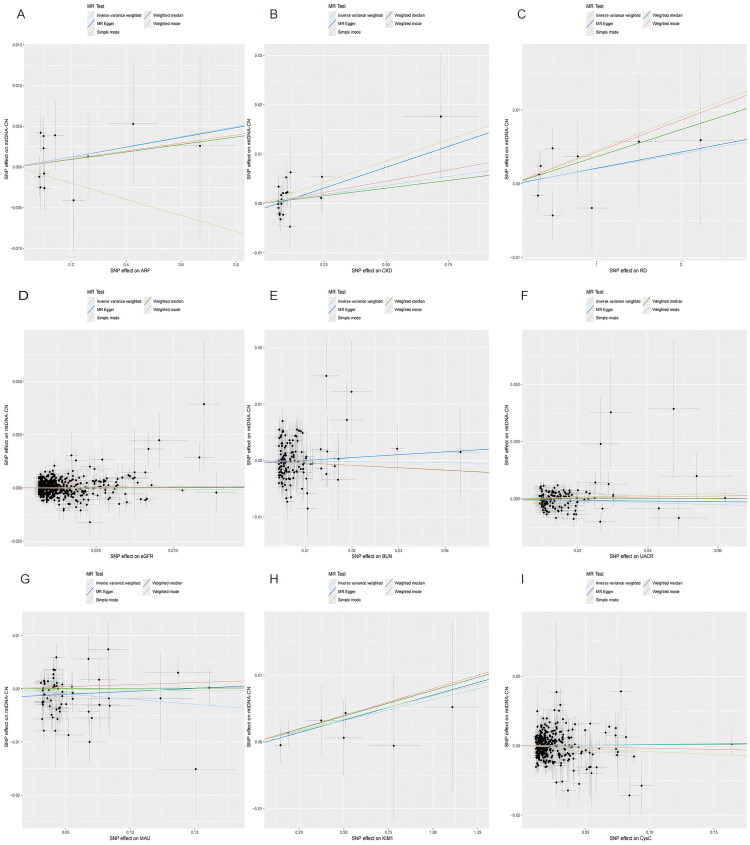
The reverse MR analyses: scatter analyses. (A) ARF, (B) CKD, (C) RD, (D) eGFR (E) BUN, (F) UACR, (G) MAU, (H) KIM-1, (I) Cys C. The four methods applied in the current manuscript were all depicted. Lines in black, red, green, and blue represent IVW, MR‐egger, weighted median, and weight mode methods.

In the sensitivity analysis, there was no significant level of pleiotropy for any of the variants (*p* > 0.05). The heterogeneity of eGFR, BUN, UACR, MAU, and CysC for mtDNA-CN was statistically significant according to the Cochrane’s Q test. Figure 6, Supplementary Figures S6 and S7 summarize the results of the sensitivity analysis (scatter plots, funnel plots, and leave-one-out analysis). Results from verification analyses aligned with current findings, demonstrating their robustness (Supplementary Tables S7 and S8, Figure S8, S9 and S10).

### MVMR analysis

The MVMR analysis showed that after integrating multivariable genetic instruments for blood cell counts (lymphocyte count, monocyte count, platelet count) and blood lipids (total cholesterol, total triglycerides, apolipoprotein A1, apolipoprotein B, apolipoprotein E), genetically predicted mtDNA-CN retained an independent causal effect on eGFR. However, after adjusting for neutrophil count, the previously observed association became non-significant. Despite this statistical significance, the confidence interval of the OR value included 1, suggesting that the practical impact of this result may be small or unstable (Supplementary Table S9).

## Discussion

This study represents the first investigation to conduct a causal association analysis between mtDNA-CN and renal function. Our research revealed a positive causal association between genetically predicted mtDNA-CN and eGFR; specifically, a decrease in mtDNA-CN is associated with an elevated risk of eGFR decline. This association was also confirmed in the MVMR analysis, where the same correlation was observed even after adjusting for several key confounding factors, including blood cell counts and blood lipids. In reverse MR analysis, the UACR and MAU demonstrated potential causal effects on mtDNA-CN, as the p-values for these two outcomes did not reach the significance threshold after multiple testing corrections, indicating the possibility of false positives and necessitating further research to establish a definitive causal relationship. The MR analysis results obtained using the two independent mtDNA-CN instrumental variables were consistent, suggesting the robustness of the findings.

In recent years, research on the relationship between mitochondrial function and kidney disease has increased gradually. Observational studies have reported controversial results regarding the association between the mtDNA-CN and renal function [48]. In a prospective study by William et al. [16] reported that a lower mtDNA-CN was associated with a lower eGFR and higher UACR, and for every one standard deviation increase in mtDNA-CN, the risk of CKD progression decreased by 10%-11%. Tewari et al. [49] confirmed that participants with a low mtDNA-CN experienced a greater decline in eGFR and had an increased risk of developing CKD and proteinuria over a five-year follow-up of a drug-using population (*N* = 946). Another study of a Chinese community-based cohort indicates that increased mitochondrial mtDNA-CN in peripheral blood is significantly linked to a decreased risk of CKD among older adults [50]. However, other studies have shown different associations between mtDNA-CN and various outcomes of kidney injury. In a large cohort study by Vasantha Jotwani et al. [51] from the UBK, after adjusting for multiple phenotypes in mtDNA-CN, they found that mtDNA haplotypes were significantly associated only with eGFR (*p* = 2.8 × 10^−12^), but not with end-stage renal disease, AKI, or MAU. Vasantha et al. [52] observed that a higher preoperative mtDNA-CN was associated with a lower risk of postoperative AKI in adults undergoing cardiac surgery; however, there was no significant association with CKD or KIM-1. These studies emphasize the critical role of mtDNA-CN in the development of kidney injury. To further clarify the causal relationship between the two, we adopted a genetic variation approach adjusted for inverse causality and confounding bias, and conducted a two-sample MR analysis using randomly assigned SNPs. To comprehensively assess kidney injury progression, we included not only conventional renal function markers (eGFR, BUN, and CysC), but also a broader range of renal function phenotypes, including indicators related to acute kidney injury (NGAL and KIM-1), markers of glomerular permeability (MAU and UACR), dynamic markers of kidney injury (CKDi25 and Rapid3), stages of kidney injury progression (ARF and CKD), and end-stage renal dialysis status The results revealed a significant positive causal relationship between mtDNA-CN and the continuous variable eGFR (OR = 1.006, *p* = 0.003). Although there was heterogeneity in the MR analysis results for both (*p* = 0.026), we used random effects inverse variance weighting as the primary analytical method, based on large-sample GWAS data. Therefore, heterogeneity is acceptable and does not affect the causal relationship. The MVMR analysis revealed that after adjusting for neutrophil count, the previously positive result turned negative, indicating that neutrophil count likely acted as a confounding factor in the original analysis. When Chong et al. adjusted mtDNA-CN using AutoMito, they similarly found that all types of cell counts were significantly associated with mtDNA-CN, with neutrophil count (*β* = −0.31; *p* < 2.23^−308^) being the strongest predictor of mtDNA-CN levels, explaining more variance (9.9%) than the combined counts of leukocytes and platelets [30]. Neutrophil count may reduce the direct effect of mtDNA-CN on eGFR through its influence on eGFR pathways. Our finding suggests that mtDNA-CN may exert a subtle but detectable genetic causal effect on renal function. However, the small effect size limits its direct application as an independent clinical biomarker. Therefore, mtDNA-CN may be more suitable as an auxiliary indicator in the comprehensive assessment of kidney injury rather than a primary diagnostic tool. Moreover, this study is the first to provide genetic evidence, laying the foundation for the development of tissue-specific mitochondrial biomarkers, which could represent a breakthrough in precision medicine for kidney disease. Future studies may need to more precisely disentangle these variables and explore their independent effects on renal function.

However, the pathophysiological mechanisms underlying this association remains unclear. The kidney is a high-energy-demand organ, second only to the heart in mitochondrial count and oxygen consumption [53]. It accounts for approximately 7% of the body’s daily ATP energy expenditure [54]. Mitochondria, as the cell’s energy factories, play a critical role in maintaining the physiological functions of kidney cells, particularly renal tubular epithelial cells and podocytes. The role of mitochondrial damage in kidney disease progression is increasingly recognized, as it significantly impacts the function of these key cells through multiple mechanisms, including disrupted energy metabolism, induced oxidative stress, promoted inflammation, and autophagy. A reduction in mtDNA-CN decreases the ability to synthesize oxidative phosphorylation-related proteins in the mitochondria, thus affecting ATP synthesis [55]. This may impair the normal function of renal tubular epithelial cells and their urine-concentrating ability, leading to a decline in renal function [56]. Previous studies have shown that mtDNA-CN in the kidneys of diabetic mice is reduced, accompanied by a downregulation of ATP production [57]. mtDNA depletion also exacerbates podocyte injury and depletion [58]. Impaired mtDNA replication in podocytes is one of the key factors contributing to renal injury in diabetic kidney disease, closely associated with mitochondrial energy metabolism disorders [59]. Additionally, the kidney, rich in mitochondria where redox reactions occur, is particularly susceptible to oxidative stress, reduced mtDNA levels can trigger intracellular oxidative stress and inflammatory responses, increasing the production of reactive oxygen species (ROS) [60]. Excessive ROS can damage cell membranes, proteins, and DNA, further leading to oxidative damage in renal tissue, exacerbating inflammation and fibrosis [61,62]. In renal fibrosis models, a decrease in mtDNA copy number is often accompanied by mitochondrial dysfunction and oxidative stress [63]. Zhao et al. found that mitochondrial ROS mediates mtDNA-related renal injury by inhibiting energy metabolism and promoting cytokine release [61]. Furthermore, a reduction in mtDNA copy number often indicates the progression of aging [64]. Milenkovic et al. reported that mice with mitochondrial DNA depletion are more prone to renal inflammation, glomerular changes, and severe chronic progressive kidney disease in old age [65]. Mitochondrial DNA defects can trigger immune responses, leading to age-related progressive pathological damage in the kidneys. With increasing age, the mtDNA content in renal tubules decreases, consistent with the gradual decline in renal function [66]. Changes in the mtDNA-CN are directly related to energy production and precede the development of various acute and chronic diseases. Additionally, mtDNA-CN serves as a DNA-based biomarker that is sufficiently stable in the internal environment of the body and can be easily obtained from various bodily fluids, including the blood. This could be accurately measured by qPCR [67,68]. Therefore, in view of the association between mtDNA-CN and eGFR, predicting the progression of kidney injury by detecting changes in blood mtDNA-CN is a promising approach. Moreover, targeting the mitochondrial copy number could be a promising therapeutic strategy for kidney diseases [69].

This study also explored the causal relationship between renal function and mtDNA-CN, using reverse MR analysis. The pathological processes of kidney disease typically involve factors such as oxidative stress, metabolic dysregulation, and inflammation, which may further influence the mtDNA-CN [61]. Preliminary results suggested a statistical association between UACR or MAU and mtDNA-CN. However, after multiple testing corrections, this association was no longer significant, indicating these findings should be regarded as hypothesis-generating rather than definitive causal evidence. Future studies with larger sample sizes and more precise renal function phenotype data are needed to validate these preliminary findings.

Our study has several strengths. We used the two-sample MR analysis method to assess the association between exposure and outcomes from a genetic variation perspective, minimizing the risk of inverse causality and confounding bias inherent in observational studies and providing stronger evidence for causal inference. We also conducted MVMR analysis to avoid false-positive inferences and further adjusted for potential confounding factors to ensure careful interpretation of the results. MR analysis using large-scale GWAS datasets improves the statistical power of causal relationship assessments and enhances the reliability of results. Furthermore, this study is the first to investigate the causal relationship between mtDNA-CN and renal function using large-sample GWAS data from two different sources, including a broader range of renal function phenotypes, offering a comprehensive analysis of the impact of mtDNA-CN on the progression of kidney injury. This study not only reveals the potential genetic mechanisms of mtDNA-CN in kidney disease but also provides a basis for the discovery of new biomarkers.

However, our study had some limitations. The study primarily relies on GWAS data from UK Biobank and CKDGen, which are predominantly based on European populations, limiting the generalizability of the results to Asian, African, or Hispanic populations. Differences in genetic background, environmental factors, and racial variations in kidney disease risk may influence the association between mtDNA-CN and renal function. To address this limitation, we recommend conducting multicenter GWAS studies, establishing regional collaborations, and developing genetic instrumental variables tailored for non-European populations. Conducting similar bidirectional MR studies in Asian (e.g., Chinese, Japanese), African, and Hispanic populations to validate the causal role of mtDNA-CN would lay the foundation for future multicenter collaborative efforts. Second, changes in mtDNA in whole blood may reflect the overall metabolic status of the body, and its specificity is not as high as that of mtDNA in the urine or kidney tissue. Recent studies have found a strong correlation between urinary mtDNA (UmtDNA) and kidney injury [70–72], suggesting that UmtDNA may have higher sensitivity and specificity for predicting kidney injury outcomes. Compared to whole-blood mtDNA-CN, UmtDNA may more directly reflect the mitochondrial status of renal tissue, as it originates from renal cell shedding or mitochondrial release. Its advantages as a tissue-specific marker include: noninvasive collection, potential for greater sensitivity in detecting early mitochondrial changes associated with kidney injury and reduced interference from systemic confounding factors (e.g., inflammatory cells in blood). If tissue-specific measurements (e.g., urinary or renal biopsy mtDNA) are used, the association between mtDNA-CN and eGFR may be stronger, as whole-blood mtDNA-CN could be diluted by non-renal-specific factors. In the future, we will further explore the relationship between UmtDNA-CN and renal function. Additionally, the overlap in GWAS data samples between exposure and outcomes could affect the independence of the results. However, we used strong instrumental variables with F-statistics >10 in the MR analysis, minimizing the potential bias in effect estimation caused by sample overlap.

In conclusion, although our study found that the MR analysis of mtDNA-CN on eGFR yielded statistically significant results, it cannot be overlooked that neutrophils, as a potential confounding factor, attenuated the causal association between the two. The causal relationships of UACR and MAU with mtDNA-CN remain unclear. These findings have potential clinical value, highlighting the critical role of mitochondria in the progression of kidney injury, and future research is needed to validate these observations and elucidate the underlying mechanisms.

## Supplementary Material

Figure S3 forward MR analysis on scatter from validation analysis using mtDNA copy number by Longchamps.tif

Figure S4 forward MR analysis on funnel from validation analysis using mtDNA copy number by Longchamps.tif

Table S6 MR analysis results of reverse MR analysis.xlsx

Figure S8 reverse MR analysis on scatte from validation analysis using mtDNA copy number by Longchamps.tif

Table S4 MR analysis results of forward MR analysis from validation analysis using mtDNA copy number by Longchamps.xlsx

Figure S7 reverse MR analysis on leave one out.tif

Table S2 MR analysis results of forward MR analysis.xlsx

Figure S9 reverse MR analysis on funnel from validation analysis using mtDNA copy number by Longchamps.tif

Table S7 Instrumental variables used in reverse MR analysis from validation analysis using mtDNA copy number by Longchamps.xlsx

Table S8 MR analysis results of reverse MR analysis based from validation analysis using mtDNA copy number by Longchamps.xlsx

Figure S2 forward MR analysis on leave one out.tif

Table S3 Instrumental variables used in forward MR analysis from validation analysis using mtDNA copy number by Longchamps.xlsx

Figure S10 reverse MR analysis on leave one out from validation analysis using mtDNA copy number by Longchamps.tif

Table S5 Instrumental variables used in reverse MR analysis.xlsx

Figure S5 forward MR analysis on leave one out from validation analysis using mtDNA copy number by Longchamps.tif

Figure S1 forward MR analysis on funnel.tif

Table S9 MVMR results analysis for association of mtDNA_CN and eGFR.xlsx

Figure S6 reverse MR analysis on funnel.tif

Table S1 Instrumental variables used in forward MR analysis.xlsx
