# Supplementary figures and images for: Mitochondrial DNA copy number as a genetic determinant of renal function: insights from bidirectional Mendelian randomization
Source: Ren Fail. 2025 Aug 10;47(1):2542522. doi: 10.1080/0886022X.2025.2542522 (PMC12340955; doi:10.1080/0886022X.2025.2542522)

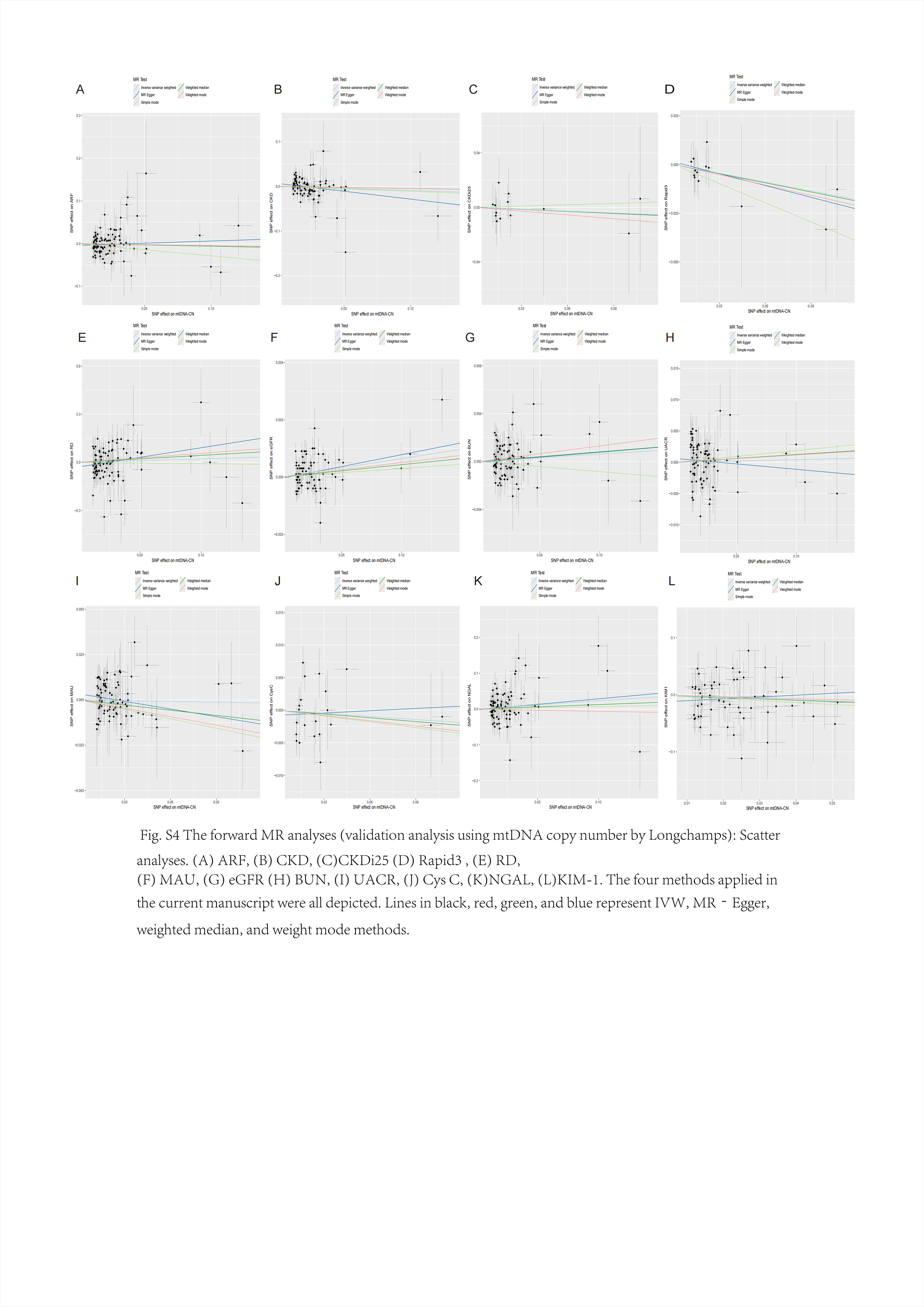

Supplement: Figure S3 forward MR analysis on scatter from validation analysis using mtDNA copy number by Longchamps.tif [file IRNF_A_2542522_SM9415.tif]

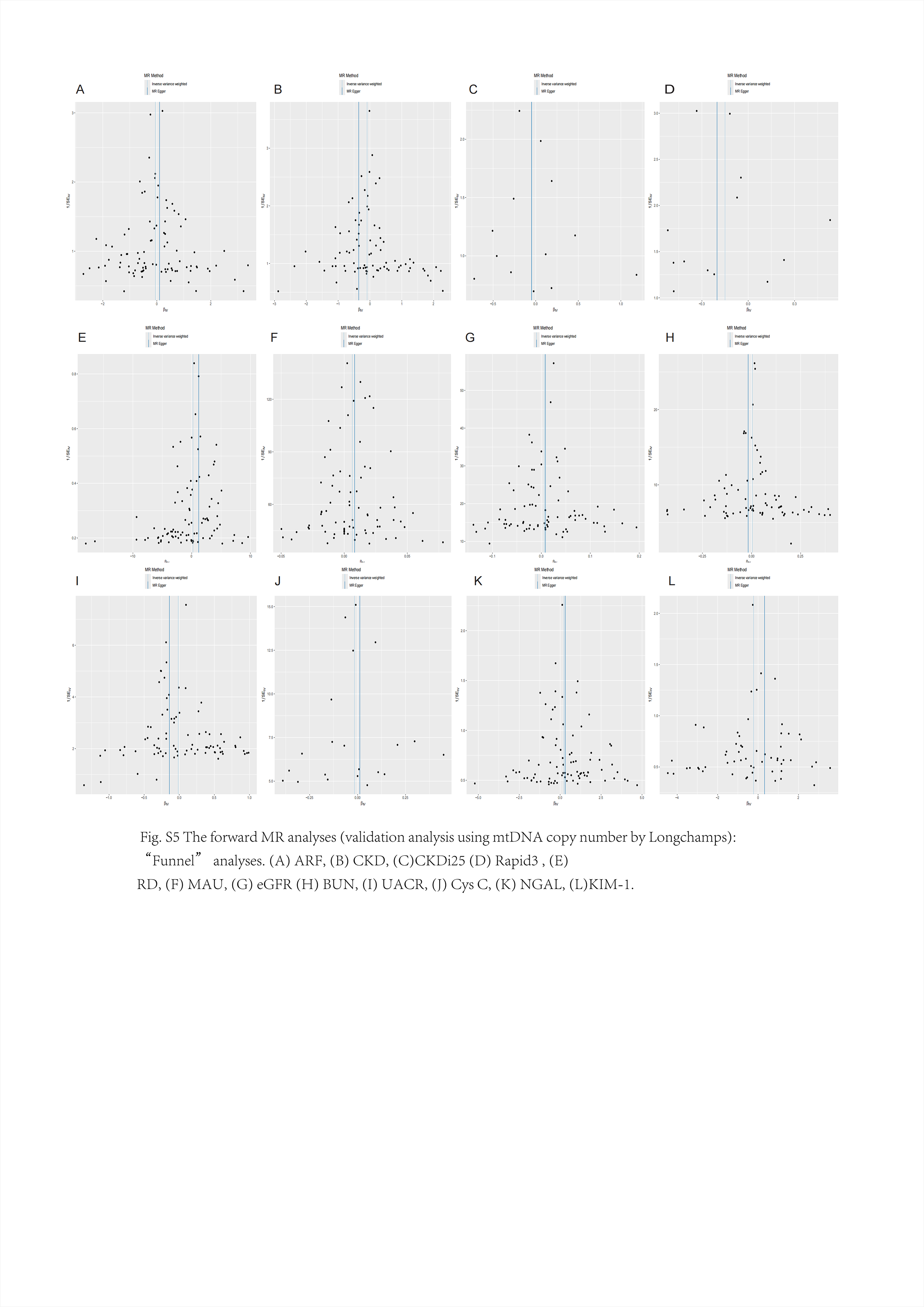

Supplement: Figure S4 forward MR analysis on funnel from validation analysis using mtDNA copy number by Longchamps.tif [file IRNF_A_2542522_SM9414.tif]

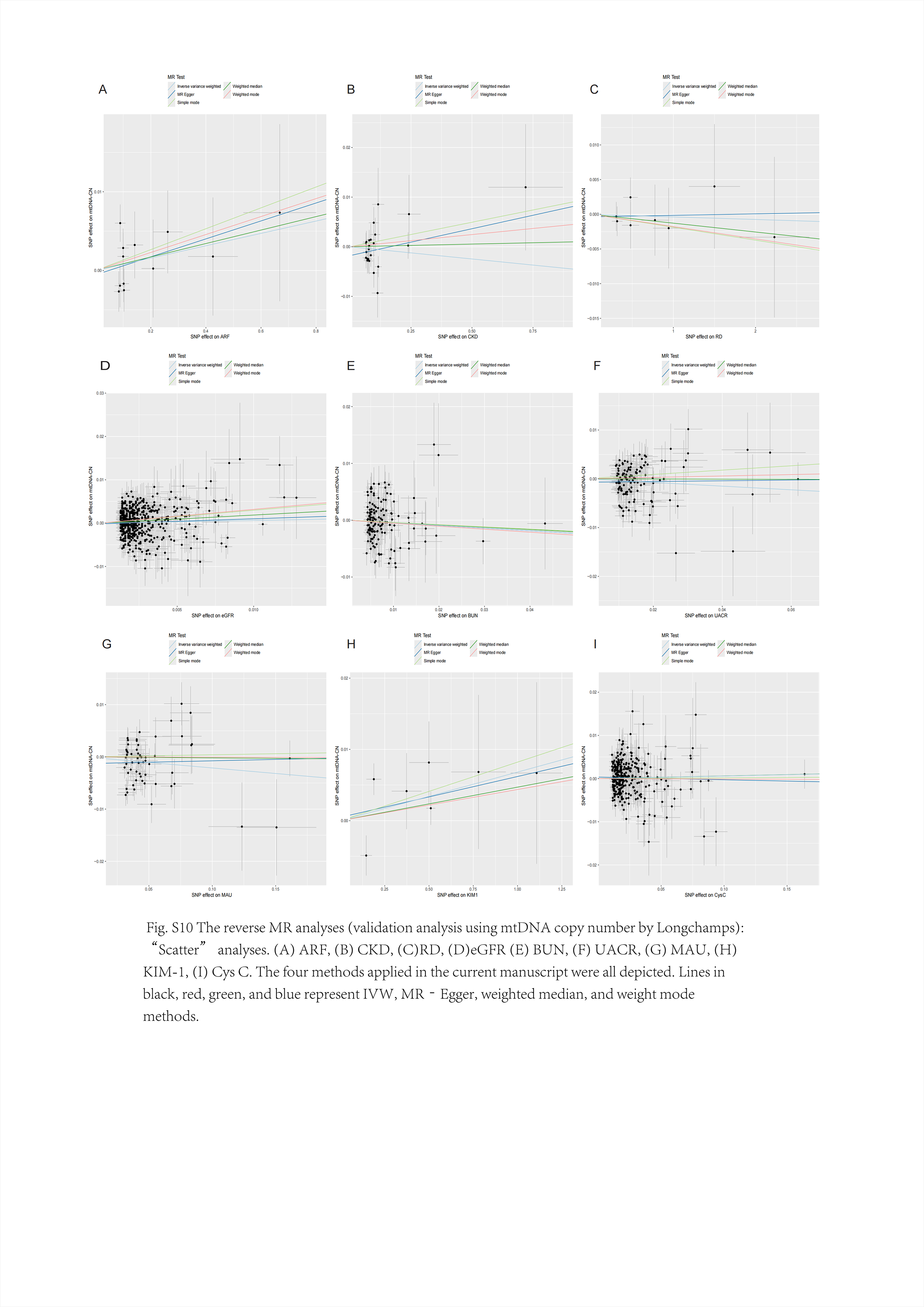

Supplement: Figure S8 reverse MR analysis on scatte from validation analysis using mtDNA copy number by Longchamps.tif [file IRNF_A_2542522_SM9412.tif]

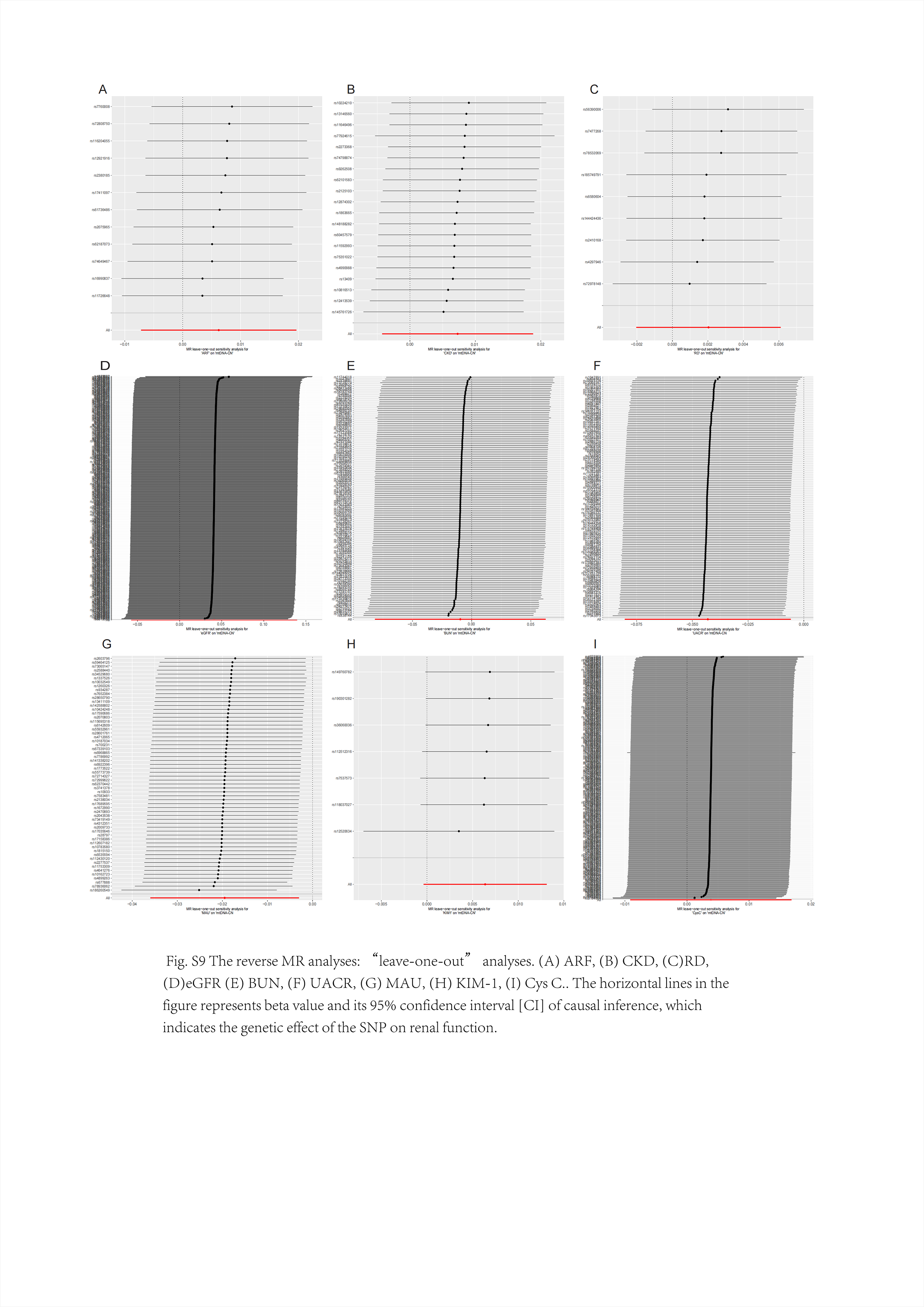

Supplement: Figure S7 reverse MR analysis on leave one out.tif [file IRNF_A_2542522_SM9410.tif]

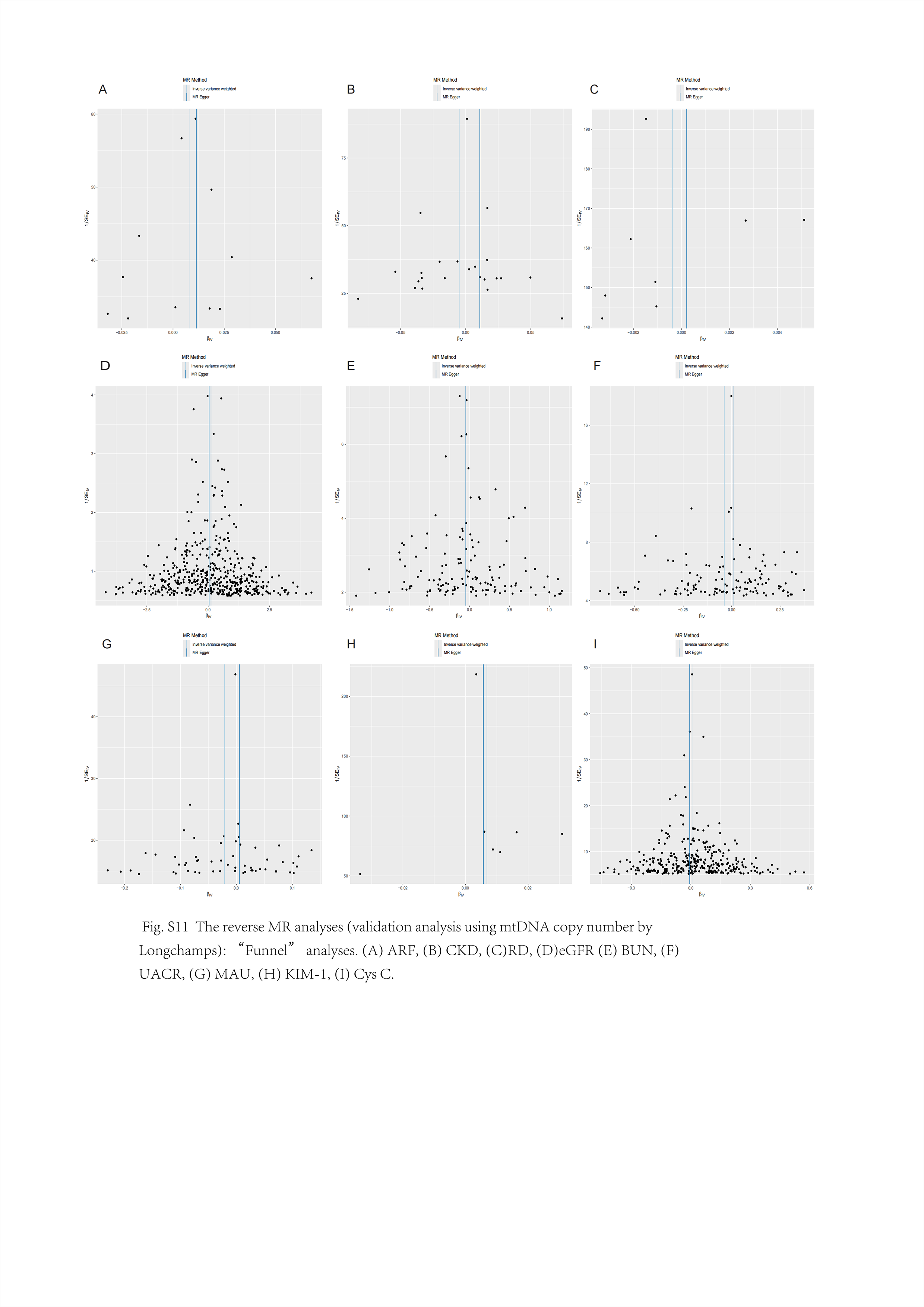

Supplement: Figure S9 reverse MR analysis on funnel from validation analysis using mtDNA copy number by Longchamps.tif [file IRNF_A_2542522_SM9407.tif]

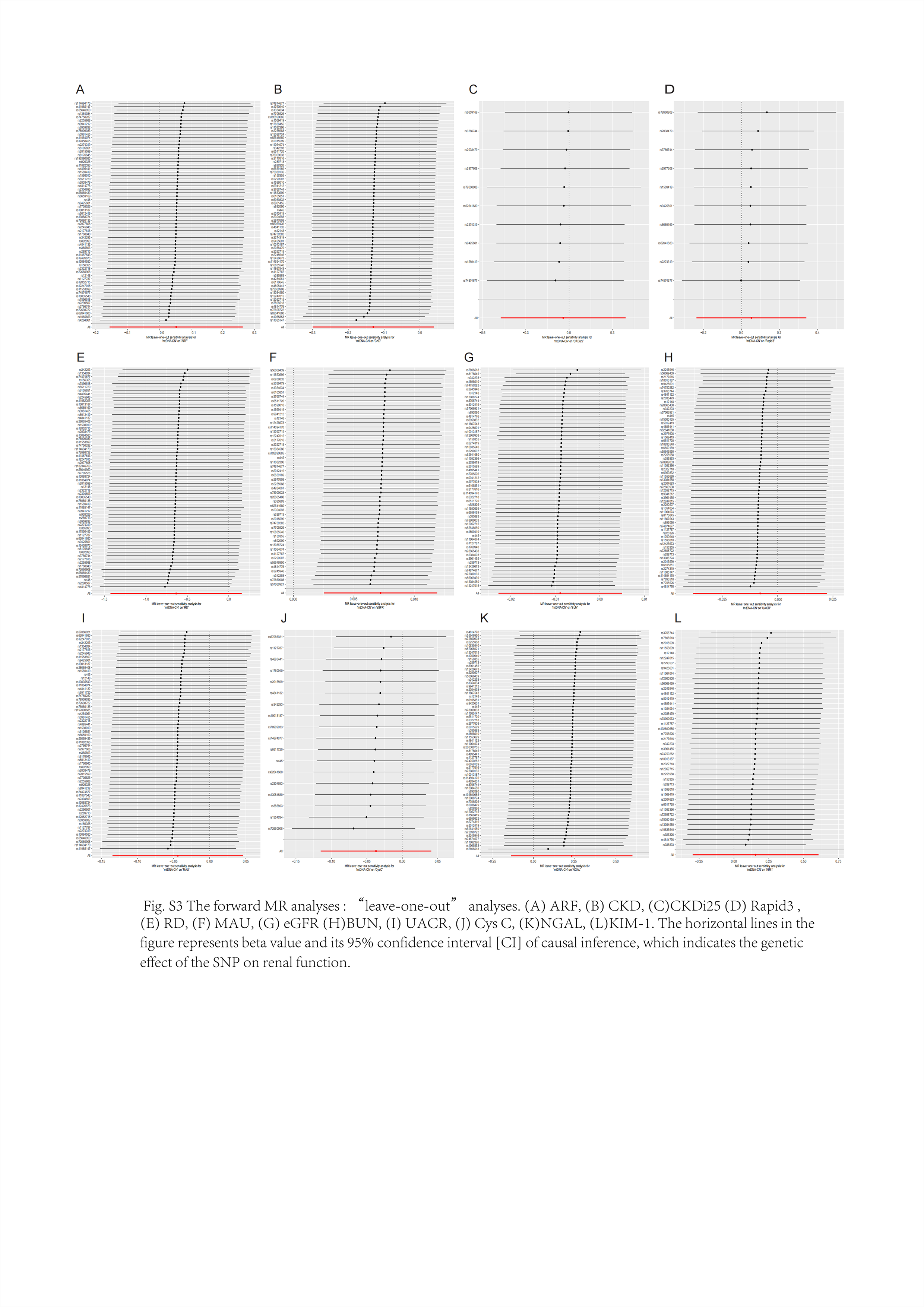

Supplement: Figure S2 forward MR analysis on leave one out.tif [file IRNF_A_2542522_SM9404.tif]

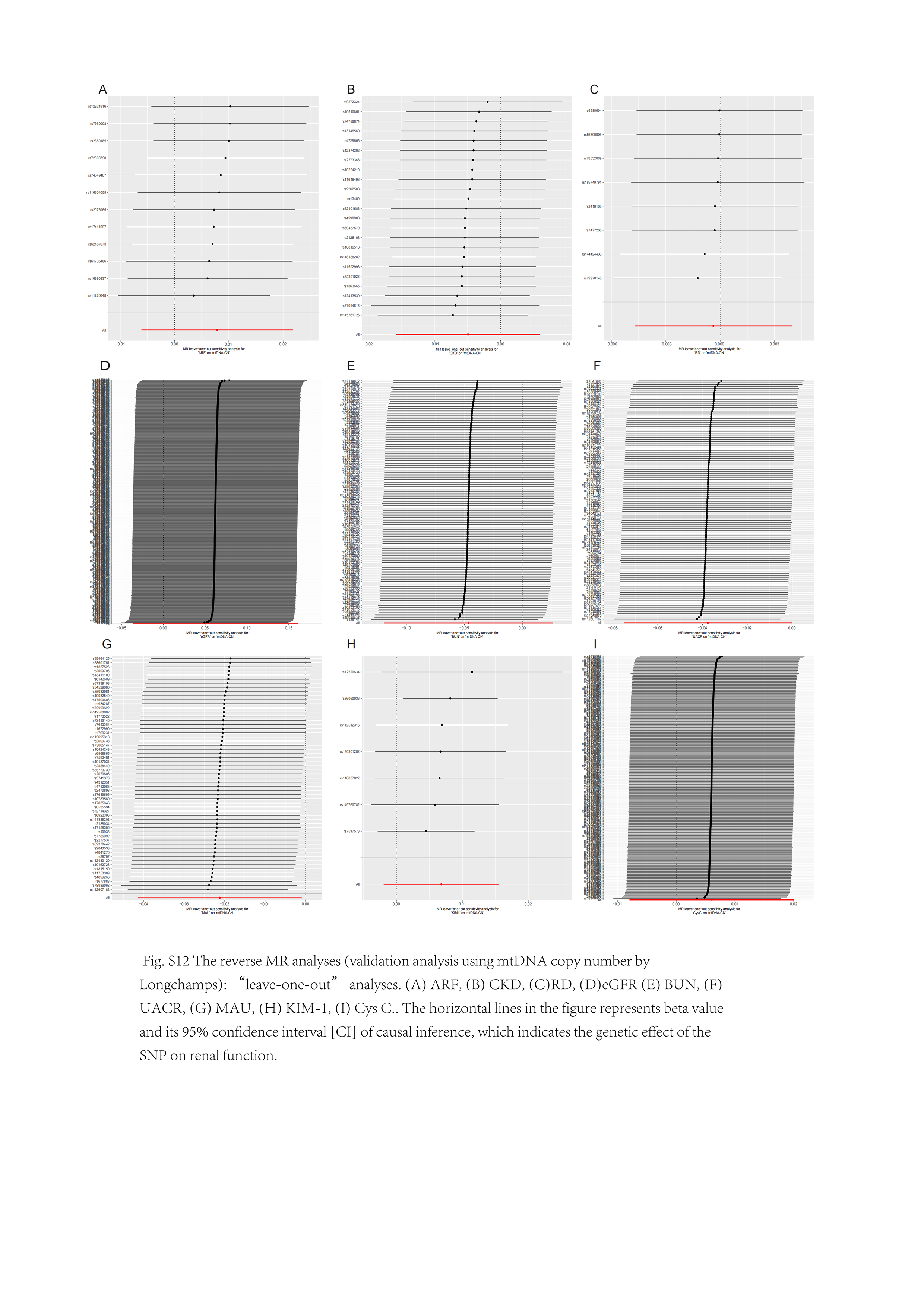

Supplement: Figure S10 reverse MR analysis on leave one out from validation analysis using mtDNA copy number by Longchamps.tif [file IRNF_A_2542522_SM9402.tif]

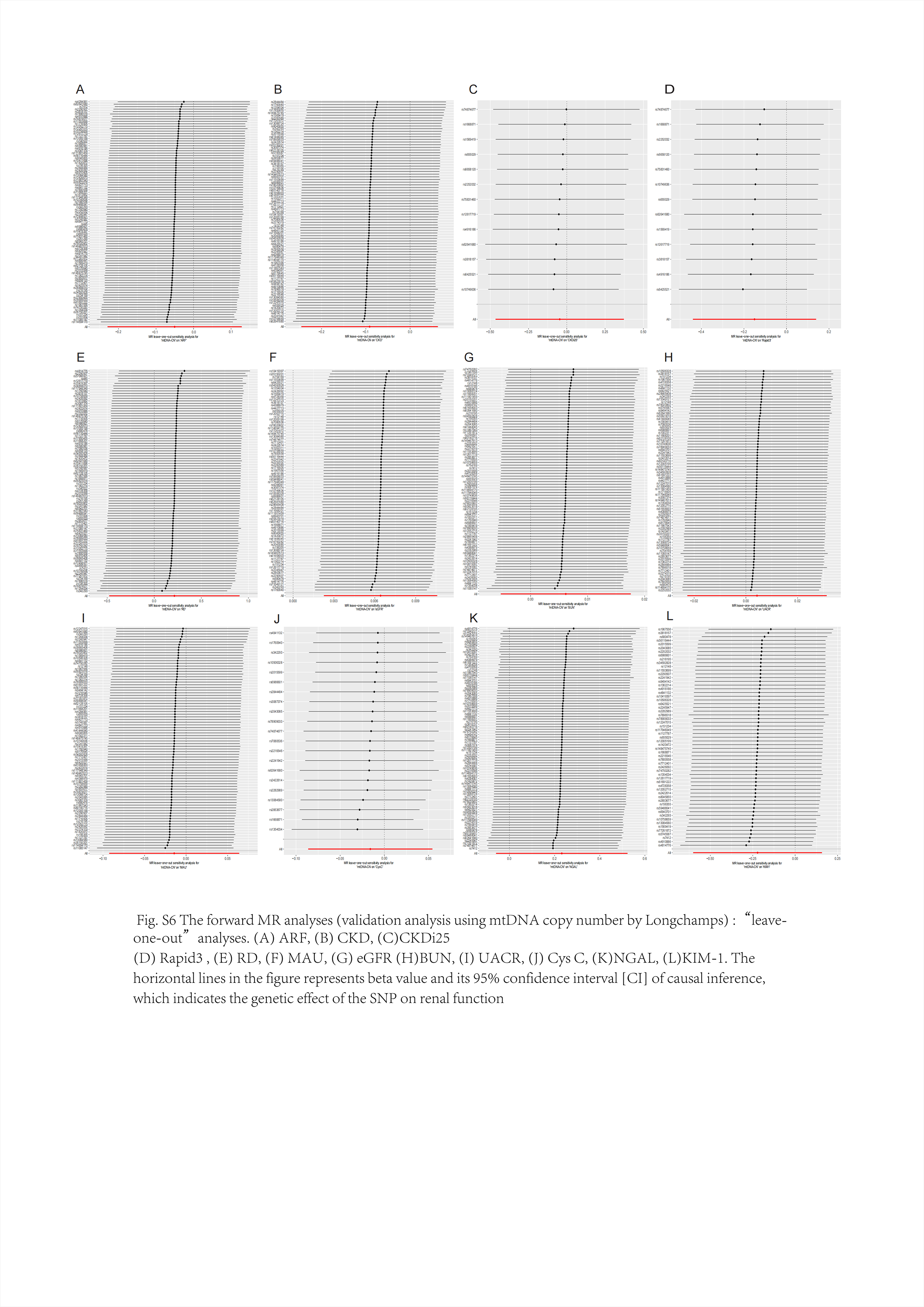

Supplement: Figure S5 forward MR analysis on leave one out from validation analysis using mtDNA copy number by Longchamps.tif [file IRNF_A_2542522_SM9400.tif]

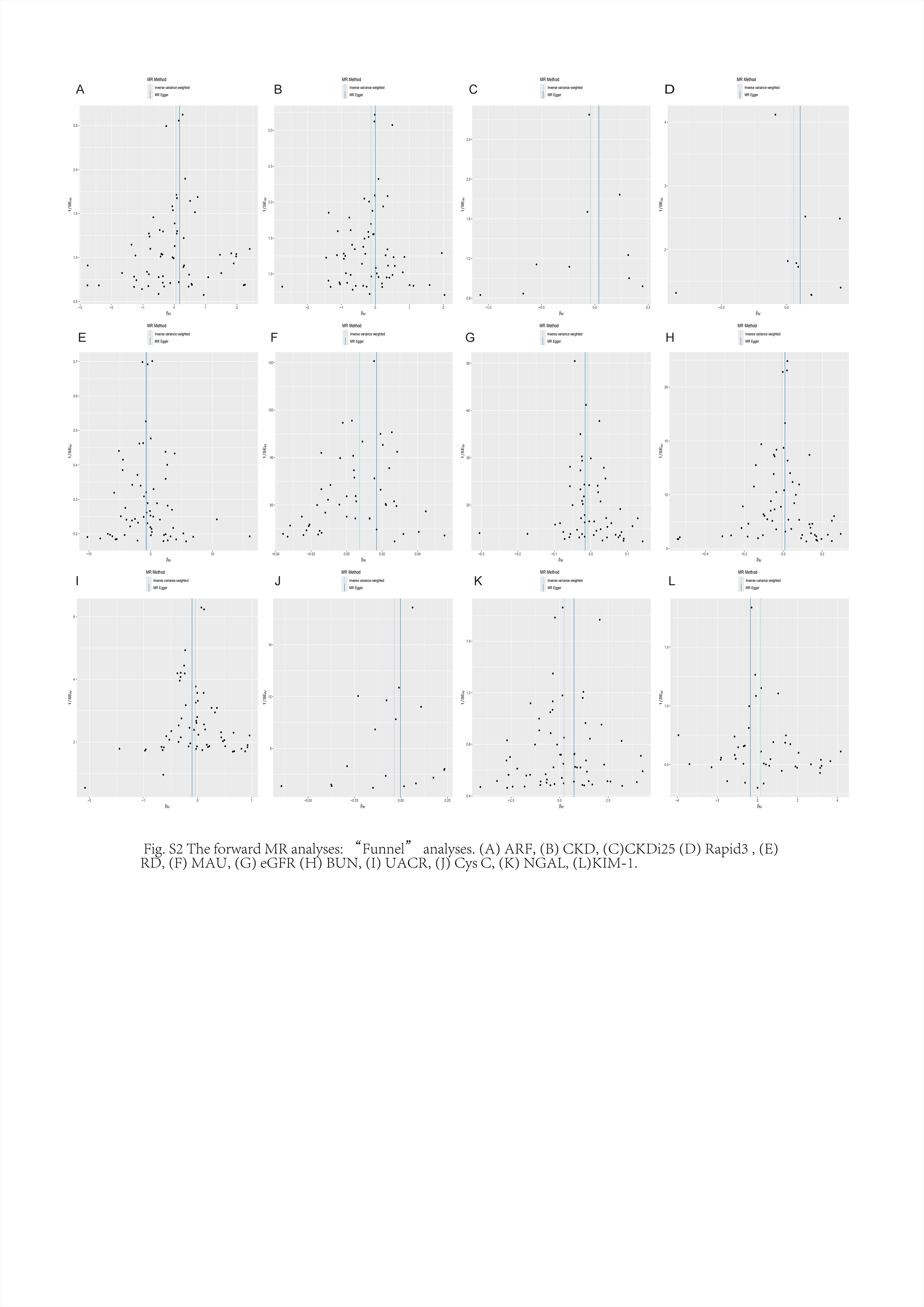

Supplement: Figure S1 forward MR analysis on funnel.tif [file IRNF_A_2542522_SM9399.tif]

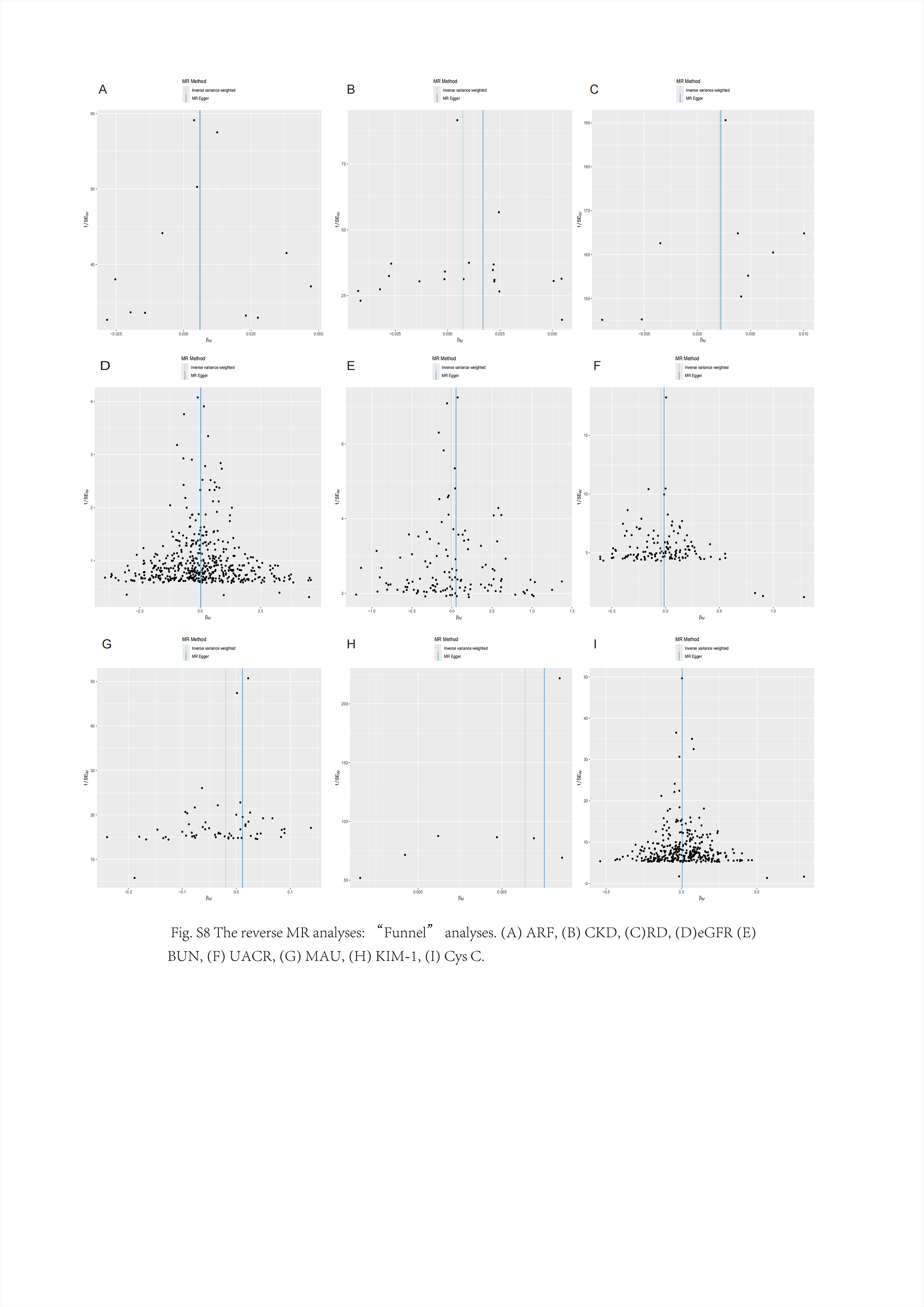

Supplement: Figure S6 reverse MR analysis on funnel.tif [file IRNF_A_2542522_SM9397.tif]
